# CLCuMuV-Induced Autophagy Restricts Viral Accumulation in the MED Cryptic Species of *Bemisia tabaci*

**DOI:** 10.3390/insects17080862

**Published:** 2026-08-19

**Authors:** Yanbo Jia, Qingxing Shi, Jie Chen, Guojun Qi, Ting Chen

**Affiliations:** Guangdong Provincial Key Laboratory of High Technology for Plant Protection, Plant Protection Research Institute, Guangdong Academy of Agricultural Sciences, Guangzhou 510640, China; jyb0202@stu.scau.edu.cn (Y.J.); shiqingxing@gdaas.cn (Q.S.); chenjie@gdaas.cn (J.C.)

**Keywords:** autophagy, DNA virus, begomovirus, insect vector, whitefly

## Abstract

Autophagy is a conserved cellular degradation pathway that plays important roles in insect–virus interactions. In the MED cryptic species of *Bemisia tabaci*, cotton leaf curl Multan virus (CLCuMuV) can persist upon acquisition, yet this vector fails to transmit the virus. However, whether autophagy contributes to this antiviral response in MED whiteflies remains unknown. In this study, we show that CLCuMuV acquisition activates autophagy in MED whiteflies, as evidenced by increased LC3-I to LC3-II conversion, enhanced LC3-positive puncta formation in the gut, and dynamic transcriptional regulation of multiple autophagy-related genes. Functional investigations using RNAi-mediated silencing of *Atg3* and *Atg9*, two essential autophagy genes, revealed that their knockdown elevated viral DNA accumulation by 1.4-fold at 48 h post-acquisition. Consistently, pharmacological blockade of autophagic flux with bafilomycin A1 led to a 1.0-, 2.2-, and 1.0-fold increase in viral loads across 24, 48, and 72 h post-acquisition, respectively, whereas treatment with rapamycin, an autophagy inducer, decreased viral DNA accumulation by 46.4% and 33.3% relative to control levels at 24 and 48 h post-acquisition, respectively. Together, these loss- and gain-of-function experiments demonstrate that autophagy functions as a host restriction mechanism that limits CLCuMuV persistence in MED whiteflies, providing molecular insights into the immune competence of this non-vector species.

## Simple Summary

Cotton leaf curl Multan virus is an important plant virus that causes severe cotton leaf curl disease. The MED cryptic species of whiteflies can acquire this virus when feeding on infected plants, yet they fail to transmit it—a puzzle that prompted us to investigate how their bodies respond to viral infection. In this study, we examined whether the virus activates autophagy, a natural recycling and degradation process inside cells. We found that autophagy was activated in whiteflies, as evidenced by increased levels of autophagy-related proteins and genes. When key autophagy genes were silenced, or the process was chemically blocked, more viral DNA accumulated in the insects. In contrast, when autophagy was stimulated, viral load decreased. These results suggest that autophagy acts as an active defense mechanism, helping whiteflies limit viral DNA accumulation inside their bodies. This work improves our understanding of how insect vectors defend against plant viruses and may inform future strategies for identifying factors that determine whether a whitefly can transmit a given virus.

## 1. Introduction

Plant viruses represent a major threat to global agricultural production, and many of them rely on insect vectors for efficient transmission between host plants. Among these viruses, members of the genus *Begomovirus* in the family *Geminiviridae* constitute one of the most diverse and damaging groups of plant DNA viruses [1,2]. Begomoviruses infect a wide range of plant species, including numerous staple, fiber, vegetable, and ornamental crops, and are responsible for severe yield losses, particularly in tropical and subtropical regions [3]. Under natural conditions, begomoviruses are transmitted by *Bemisia tabaci* in a persistent-circulative manner [4]. During virus acquisition, whiteflies ingest virions from the phloem sap of infected plants through their stylets. The virions subsequently pass through the alimentary canal, cross the midgut epithelial barrier, enter the hemolymph, and eventually reach the salivary glands. During subsequent feeding on healthy plants, virions are released with saliva, thereby completing horizontal transmission between plant hosts [5]. Increasing evidence indicates that begomovirus acquisition is not a passive process within the insect vector. Instead, virus acquisition and circulation can induce broad physiological, cellular, molecular, and behavioral responses in whiteflies, which may influence vector fitness, virus accumulation, and transmission efficiency [6]. However, the molecular mechanisms underlying these vector responses remain incompletely understood.

Long-term coevolution has shaped intricate interactions between begomoviruses and their whitefly vectors. Although begomovirus acquisition usually does not cause acute lethality in whiteflies, it can impose fitness costs and alter multiple biological processes in the vector. Once acquired, begomoviruses may persist in whiteflies for extended periods, and in some cases for much of the insect lifespan [7,8,9]. During circulation within the vector body, virions encounter multiple tissue barriers and cellular defense systems, which may activate innate immune responses. Comparative transcriptomic analyses have shown that infection with tomato yellow leaf curl China virus (TYLCCNV) alters the expression of genes associated with basal metabolism and immune regulation in whiteflies, including genes involved in autophagy and mitogen-activated protein kinase signaling [10,11]. Functional studies have further demonstrated that autophagy acts as an antiviral mechanism in the MEAM1 cryptic species of *B. tabaci* against tomato yellow leaf curl virus (TYLCV), suppressing viral accumulation through the degradation of viral coat protein and genomic DNA [12]. In addition to autophagy, other immune-related factors, such as the antimicrobial peptide Knottin, have also been implicated in whitefly antiviral defense, as silencing of Knottin-1 increases TYLCV accumulation in the vector [13]. These findings suggest that whiteflies possess active immune mechanisms that can regulate begomovirus persistence and accumulation.

Among these immune mechanisms, autophagy is an evolutionarily conserved intracellular degradation pathway that delivers cytoplasmic materials, protein aggregates, damaged organelles, and invading pathogens to lysosomes for degradation and recycling [14,15,16]. In insects, autophagy plays essential roles in development, stress tolerance, metabolic homeostasis, and innate immunity [17]. As an antiviral defense mechanism, autophagy can restrict viral infection by directly targeting viral proteins, viral nucleic acids, or virus-containing vesicles for degradation. However, the role of autophagy in virus–host interactions is highly context-dependent. In some systems, autophagy suppresses viral replication or transmission, whereas in others, viruses exploit autophagic membranes or autophagy-related signaling pathways to promote their own replication and spread [18,19]. For example, in MEAM1 whiteflies, TYLCV-induced autophagy restricts viral accumulation [11]; conversely, in the silkworm *Bombyx mori*, nucleopolyhedrovirus subverts the autophagic pathway to promote its own replication [18]. These contrasting outcomes highlight the complex and dynamic nature of autophagy-mediated virus–host interactions in insects.

Cotton leaf curl Multan virus (CLCuMuV), together with its associated betasatellite, is one of the major causal agents of cotton leaf curl disease, which has been devastating cotton crops in Pakistan and India for decades [20]. This virus infects a broad range of hosts in the family *Malvaceae*, including cotton (*Gossypium* spp.), okra (*Abelmoschus esculentus*), Chinese hibiscus (*Hibiscus rosa-sinensis*), and wax mallow (*Malvaviscus arboreus* var. *penduliflorus*) [20,21]. Since its first report in Guangdong Province in China, CLCuMuV has spread to several other provinces, including Guangxi, Hainan, Yunnan, and Fujian, and has recently reached Xinjiang, the country’s major cotton-producing region [22]. Its continued spread poses a serious threat to cotton production and to other economically important or ornamental host plants. Previous studies have shown that AsiaII7 and AsiaII1 cryptic species of *B. tabaci* are efficient vectors of CLCuMuV [23,24]. The Mediterranean (MED) whitefly is widely distributed in China and can acquire CLCuMuV [23,25], yet it is considered a non-vector due to its inability to transmit the virus [23]. Nevertheless, CLCuMuV can persist and accumulate within MED whiteflies upon acquisition, and this persistent infection may impose physiological costs on the vector. This raises the question of what cellular mechanisms constrain viral accumulation in this non-vector species. Our previous transcriptomic analysis of CLCuMuV-acquiring MED whiteflies identified numerous differentially expressed genes, several of which were significantly enriched in autophagy-related pathways [26]. This enrichment led us to speculate that MED-mediated autophagy might be involved in restricting viral accumulation, potentially through degradation or clearance of the virus, thereby preventing its transmission to recipient plants. However, whether this transcriptional response translates into functional autophagy activation remains unknown, and if so, whether it affects viral accumulation in MED whiteflies remains to be determined.

In this study, unlike previously characterized autophagy responses in competent whitefly–begomovirus systems, the MED–CLCuMuV association examined here enables direct assessment of viral DNA accumulation in a non-competent vector species. Specifically, we examine whether CLCuMuV acquisition triggers autophagic responses in vivo, and whether such responses contribute to restricting viral accumulation. By addressing these questions, this study provides insights into the antiviral immune mechanisms operating in a begomovirus–whitefly system in which transmission is naturally blocked, offering a unique perspective on the interplay between viral persistence and host cellular defense.

## 2. Materials and Methods

### 2.1. Insects, Plants and Viruses

Cotton plants (*Gossypium hirsutum* cv. Zhongmian-838), which are non-susceptible to CLCuMuV, were grown in pots (diameter: 10 cm; height: 12 cm) inside insect-proof cages (60 × 60 × 60 cm). All plants were maintained in a greenhouse under a long-day photoperiod (16 h light/8 h dark) at 25–27 °C and 60% relative humidity. The colony of *B. tabaci* MED cryptic species was initially collected from the tomato plants in Guangzhou City, China, in 2020 and maintained on Zhongmian-838 cotton plants in the greenhouse. To ensure the purity of the whitefly population, the *mtCOI* gene was utilized for regular monthly identification checks [27]. The infectious clones of CLCuMuV (GenBank accession number KP762786) and beta-satellite (KP762787) were obtained in the Plant Protection Research Institute at the Guangdong Academy of Agricultural Sciences, and introduced into the cotton plants (Xinhai mian-21, a susceptible variety) via agroinoculation with the GV3101 strain. Infected Xinhai mian-21 cotton plants were used for assays at 4–5 weeks post-inoculation, when typical leaf curl symptoms became apparent [28].

### 2.2. Nucleic Acid Extraction and Quantitative PCR

Non-viruliferous *B. tabaci* adults were collected from healthy plants and placed on CLCuMuV-infected plants for a 24, 48, or 72 h acquisition access period (AAP), with non-exposed adults serving as 0 h controls. Then, 80 *B. tabaci* adults were selected for each experiment. Total DNA was extracted using a DNA extraction kit (Tiangen, Beijing, China), and total RNA was extracted using TRIzol reagent (Invitrogen, Carlsbad, CA, USA) from separate aliquots of the same pooled sample. For viral DNA quantification, CLCuMuV accumulation was measured by qPCR using primers specific to the viral coat protein (CP) gene (primers listed in Table 1). For autophagy-related gene expression analysis, RNA was reverse transcribed to cDNA using a cDNA synthesis kit (TaKaRa, Shiga, Japan), and qPCR was performed on the CFX96 Real-Time PCR Detection System (Bio-Rad, Hercules, CA, USA) with SYBR green detection (Kapa Biosystems, Boston, MA, USA). The whitefly *β-actin* gene was used as the internal reference for normalization. Relative transcript levels were calculated using the 2^−ΔΔCt^ method. Each sample was analyzed in three technical repeats, and three independent biological replicates were performed for each treatment, yielding a total of 240 whiteflies (*n* = 240) analyzed per treatment.

### 2.3. Western Blot Analysis

Non-viruliferous and CLCuMuV-viruliferous whiteflies were collected separately and independently homogenized in RIPA lysis buffer (Beyotime, Shanghai, China) supplemented with a protease inhibitor cocktail (Roche, Basel, Switzerland) with protease inhibitors and analyzed by Western blotting as previously described [29]. For each sample, 100 insects were pooled per lane, 20 μg protein was loaded per lane, and three independent blots were performed. Commercial antibodies to LC3B (SIGMA-L7543, 1:1000 dilution), secondary anti-rabbit (Abcam, ab6721, 1:5000 dilution), β actin (Abcam 8226, 1:5000 dilution) were purchased from Sigma-Aldrich and Abcam, respectively. Band intensities were quantified using ImageJ 2 software (NIH, Bethesda, MD, USA), and LC3-II ratios were normalized to β-actin levels for each sample.

### 2.4. Confocal Immunofluorescence Microscopy

Whitefly midguts were dissected in phosphate-buffered saline (PBS) and fixed in 4% paraformaldehyde for 2 h at room temperature. The midguts were washed three times in TBST, permeabilized with 0.1% Triton X-100 for 1 h, rinsed again in TBST, blocked in 0.5% BSA-TBST, and subsequently incubated with primary antibody (ATG8/GABARAP; Cell Signal Technology, Danvers, MA, USA, 13733; 1:1000 dilution) overnight at 4 °C. The guts were then washed twice in TBST and incubated with secondary antibody (anti-rabbit IgG conjugate Alexa 555; Abcam, Cambridge, CA, UK, 150078; 1:5000 dilution) diluted in TBST for 2 h at room temperature. After washing three times in TBST, the samples were mounted in Fluoroshield mounting medium with DAPI (Beyotime, Shanghai, China, P0131) and imaged using a Zeiss LSM780 confocal microscope (Zeiss, Oberkochen, Germany). Three independent biological replicates were performed for each treatment, with 20 midguts examined per replicate. Acquisition settings (e.g., exposure time, laser intensity, gain) were kept identical across all groups to ensure comparability.

### 2.5. Atg3, Atg9, Silencing by RNA Interference

The dsRNA was synthesized using the T7 RiboMAX^TM^ Express RNAi System (Promega, Madison, WI, USA) with *dsAtg3*, *dsAtg9*, and *dsGfp* in Table 1. For RNA interference, the sucrose diet with 200 ng/μL *dsAtg3*, *dsAtg9* dsRNA was used as the treatment group, and the sucrose diet containing 200 ng/μL *dsGfp* was used as a negative control. First, 200 whiteflies at 3 to 5 days old were fed with *dsAtg3*, *dsAtg9*, or *dsGfp* in cylindrical containers for 24 h and then transferred to CLCuMuV-infected cotton plants to acquire the virus, respectively. After a 24, 48, and 72 h acquisition access period (AAP), the whiteflies were collected for subsequent Western blot, confocal microscopy, and qPCR analyses. All treatment groups were replicated three times.

### 2.6. Treatment with Autophagy Inhibitor and Inducer

To investigate the functional role of autophagy in CLCuMuV infection, the autophagy inhibitor bafilomycin A1 (BAF; Selleck, Houston, TX, USA, S1413) and rapamycin- an mTOR inhibitor commonly used to induce autophagy (Selleck, Houston, TX, USA, S1039) were used. Both compounds were dissolved in dimethyl sulfoxide (DMSO), and the final DMSO concentration was adjusted to 0.1% (*v*/*v*) in all treatment solutions. Adult whiteflies were first pre-treated for 24 h with 25% sucrose solution containing 10 nM BAF, 10 nM rapamycin, or an equivalent volume of DMSO (final DMSO concentration 0.1% *v*/*v*) as solvent control, in cylindrical containers (30 mm diameter). After pre-treatment, the whiteflies were transferred to virus-infected or healthy cotton plants for virus acquisition. At 24 and 48 h post-transfer, 160 whiteflies were collected from each treatment group and divided into two aliquots (80 each) for DNA and RNA/protein extraction, respectively. Viral DNA accumulation was quantified by qPCR using CLCuMuV coat protein-specific primers, and autophagy activation was evaluated by Western blot and RT-qPCR. Three independent biological replicates were performed for each treatment.

### 2.7. Statistical Analysis

Data are presented as mean ± standard error (SE). GraphPad Prism 12.0 (GraphPad Software, La Jolla, CA, USA) was used for statistical analyses; one-way ANOVA with Tukey’s HSD multiple comparison test and Student’s *t*-test was applied to compare groups. A *p*-value < 0.05 was considered statistically significant, whereas *p* < 0.01 was regarded as extremely significant.

## 3. Results

### 3.1. Autophagy Pathway Is Activated in Whiteflies in Response to CLCuMuV Infection

**Figure 1:**
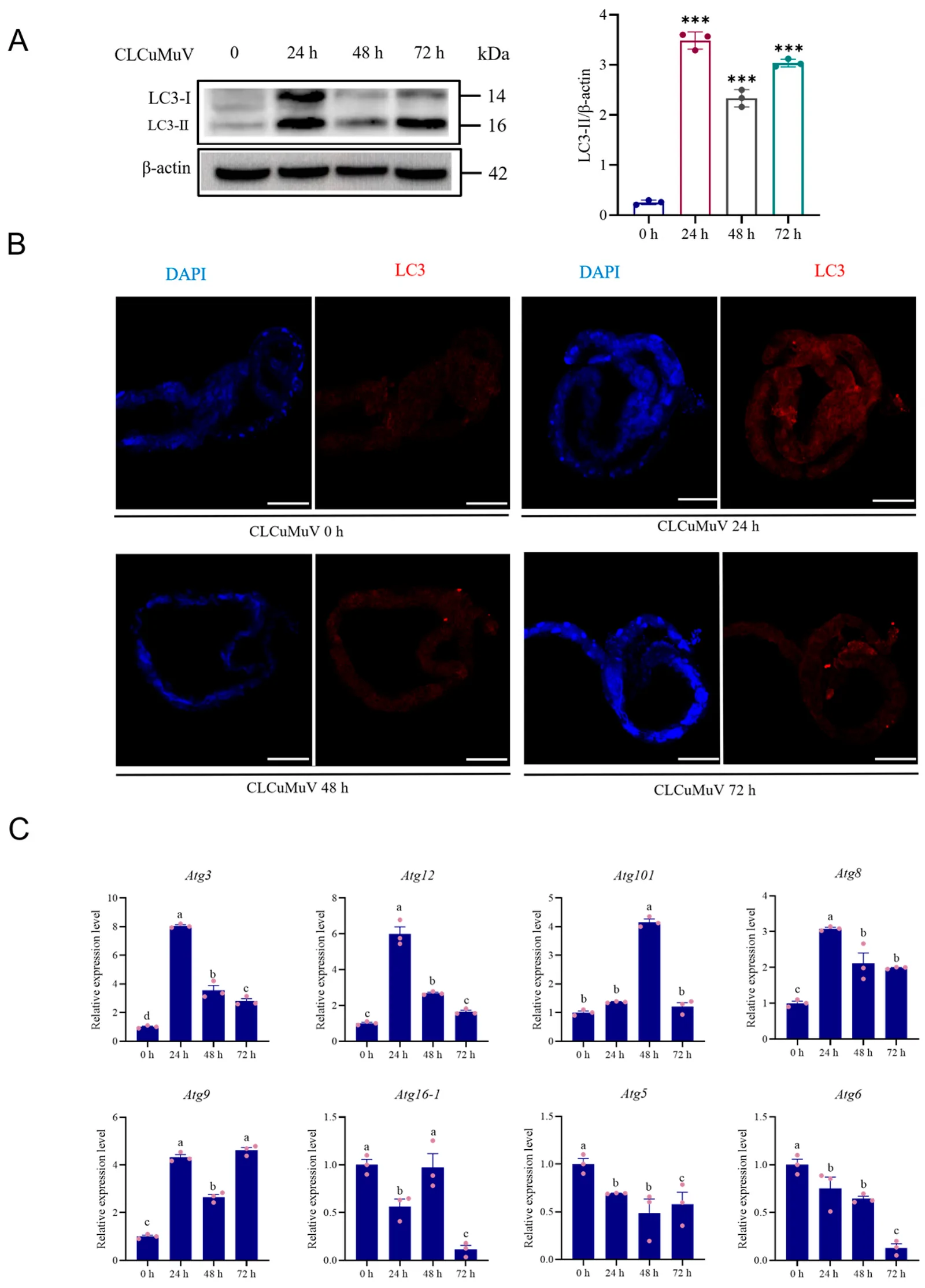
CLCuMuV infection activates autophagy in *Bemisia tabaci*. (**A**) Western blot analysis of LC3-I/LC3-II conversion expression in whiteflies at 0, 24, 48, and 72 h post-acquisition. β-actin was used as an internal control. The bar graph on the right represents the quantitative ratio of LC3-II/β-actin based on band intensities; data were analyzed by one-way ANOVA with Tukey’s HSD test, *** *p* < 0.001. (**B**) Immunofluorescence microscopy showing the distribution and puncta formation of LC3 (red) within the gut of whiteflies at the indicated time points (0, 24, 48, and 72 h). Nuclei were stained with DAPI (blue). Scale bars are indicated in the panels. (**C**) Relative mRNA expression levels of eight autophagy-related genes were determined by RT-qPCR at 0, 24, 48, and 72 h post-acquisition. Statistical significance was analyzed using one-way ANOVA followed by Tukey’s HSD test; different lowercase letters (a, b, c, d) above the columns indicate statistically significant differences at *p* < 0.05.

To investigate whether CLCuMuV infection triggers autophagic responses in MED cryptic species of *B. tabaci*, we examined the protein and mRNA expression profiles of key autophagy-related markers at different time points post-acquisition (0, 24, 48, and 72 h). First, Western blot analysis was performed to evaluate the lipidation of microtubule-associated protein 1 light chain 3 (LC3-I to LC3-II), a hallmark of autophagosome formation. The results showed that LC3-II levels were significantly elevated at 24, 48, and 72 h post-acquisition compared to uninfected controls (Figure 1A). Quantification of the LC3-II/β-actin ratio further confirmed a significant accumulation of autophagosomes at all three time points.

To further corroborate the enhanced autophagic activity, immunofluorescence microscopy was conducted to visualize the subcellular localization of LC3. As shown in Figure 1B, while faint and diffuse LC3 signals were detected in uninfected whiteflies, robust and punctate LC3-positive aggregates (red fluorescence) prominently accumulated in the cytoplasm of infected whiteflies at 24, 48, and 72 h. This intensified punctate staining pattern serves as representative qualitative evidence for elevated autophagosome formation following CLCuMuV infection.

Subsequently, the transcriptional levels of eight autophagy-related genes *Atg3*, *Atg12*, *Atg101*, *Atg8*, *Atg9*, *Atg16-1*, *Atg5*, and *Atg6* were quantified using RT-qPCR. The results revealed a dynamic and gene-specific transcriptional response (Figure 1C). Among these genes, *Atg3*, *Atg8*, *Atg9*, and *Atg12* showed early upregulation, with peak mRNA levels at 24 h post-infection followed by gradual declines at 48 and 72 h. In contrast, *Atg101* exhibited a delayed response, with its highest expression observed at a later time point (48 h). *Atg16-1* did not display the early 24 h induction pattern seen in the aforementioned genes, instead showing a relatively stable expression level throughout the time course. Meanwhile, *Atg5* and *Atg6* displayed a distinct trend of progressive downregulation, reaching their lowest levels at 72 h. Collectively, these results demonstrate that CLCuMuV infection activates cellular autophagy in MED whiteflies, as evidenced by autophagosome accumulation and the dynamic transcriptional regulation of multiple *Atg* genes, despite the downregulation of *Atg5* and *Atg6* at later time points.

### 3.2. Inhibition of Autophagy Promotes CLCuMuV Accumulation in Bemisia tabaci

**Figure 2:**
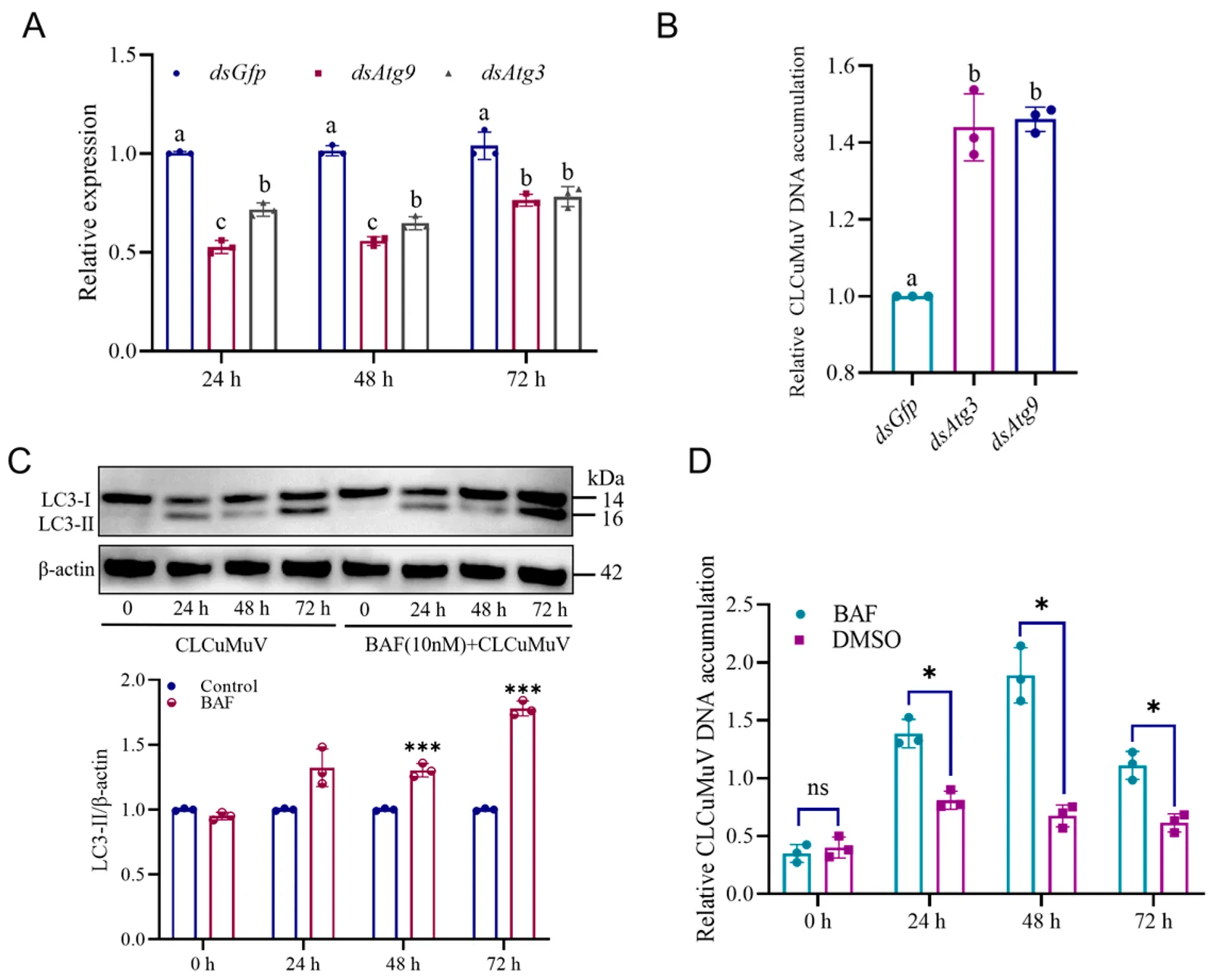
Inhibition of the autophagy pathway promotes CLCuMuV accumulation in *Bemisia tabaci*. (**A**) Validation of RNA interference (RNAi) efficiency *dsAtg9*, and *dsAtg3*. Relative mRNA expression levels of target genes were detected by RT-qPCR. Data were analyzed by one-way ANOVA with Tukey’s HSD test; different lowercase letters (a, b, c) above the columns indicate statistically significant differences at *p* < 0.05. (**B**) Effects of *Atg9* and *Atg3* knockdown on CLCuMuV DNA accumulation in whiteflies, same data analysis method as (A). (**C**) Effects of the autophagy inhibitor bafilomycin A1 (BAF) on LC3-I/LC3-II conversion. The bar graph below shows the quantitative ratio of LC3-II/β-actin based on band intensities; data were analyzed with Student’s *t*-test, *** *p* < 0.001. (**D**) Effects of blocking autophagic flux (via BAF treatment) on the relative accumulation of CLCuMuV DNA in whiteflies. Statistical significance was analyzed with Student’s *t*-test; “ns” indicates no significant difference, while asterisks indicate * *p* < 0.05.

To determine the function of autophagy in viral propagation, we investigated the effects of both genetic silencing and chemical inhibition of autophagy on CLCuMuV accumulation in the whiteflies. First, we performed RNA interference (RNAi) to knock down the highly upregulated *Atg9* and *Atg3*. qPCR analysis confirmed that the mRNA expression levels of *Atg9* and *Atg3* were significantly suppressed at 24, 48, and 72 h post-dsRNA feeding, compared with the *dsGfp*-treated control group (Figure 2A). For subsequent RNAi experiments, whiteflies were fed on dsRNA for 24 h and then transferred to virus-infected cotton plants for an additional 48 h. Notably, silencing of either *Atg9* or *Atg3* significantly increased CLCuMuV DNA accumulation, with levels rising to 1.4-fold that of the *dsGfp* control, indicating that RNAi-mediated knockdown of these autophagy components facilitates viral accumulation (Figure 2B).

Next, to verify this observation with a pharmacological approach, we treated whiteflies with bafilomycin A1 (BAF), a well-characterized lysosomal inhibitor that blocks autophagic flux by preventing autophagosome-lysosome fusion. After a 24 h exposure to BAF, whiteflies were fed on virus-infected cotton plants for 0, 24, 48, and 72 h, and the impact of BAF on autophagy was assessed using Western blot analysis. Western blot analysis revealed that LC3-II levels were drastically elevated in the BAF-treated group at 24, 48, and 72 h post-acquisition compared to the virus-only control group (Figure 2C). This accumulation of LC3-II is consistent with the expected effect of BAF-mediated blockade of autophagosome-lysosome fusion. Importantly, when autophagic flux was blocked by BAF, the relative accumulation of CLCuMuV DNA was 1.0-, 2.2-, and 1.0- fold higher than in the control group at 24, 48, and 72 h post-acquisition (Figure 2D). Collectively, both genetic knockdown and chemical inhibition of autophagy significantly increased viral load in the whitefly. To determine whether the elevated viral load caused by autophagy suppression could restore transmissibility, we performed transmission assays using *Atg3-* and *Atg9*-silenced whiteflies on healthy cotton plants. At 30 days post-inoculation, all recipient plants remained asymptomatic, and neither conventional PCR nor qPCR detected the presence of the CLCuMuV DNA in any of the tested samples (Supplementary Table S1, Figure S1), indicating that autophagy inhibition alone is insufficient to overcome the transmission barrier in MED whiteflies.

### 3.3. Activation of Autophagy Restricts CLCuMuV Accumulation

**Figure 3:**
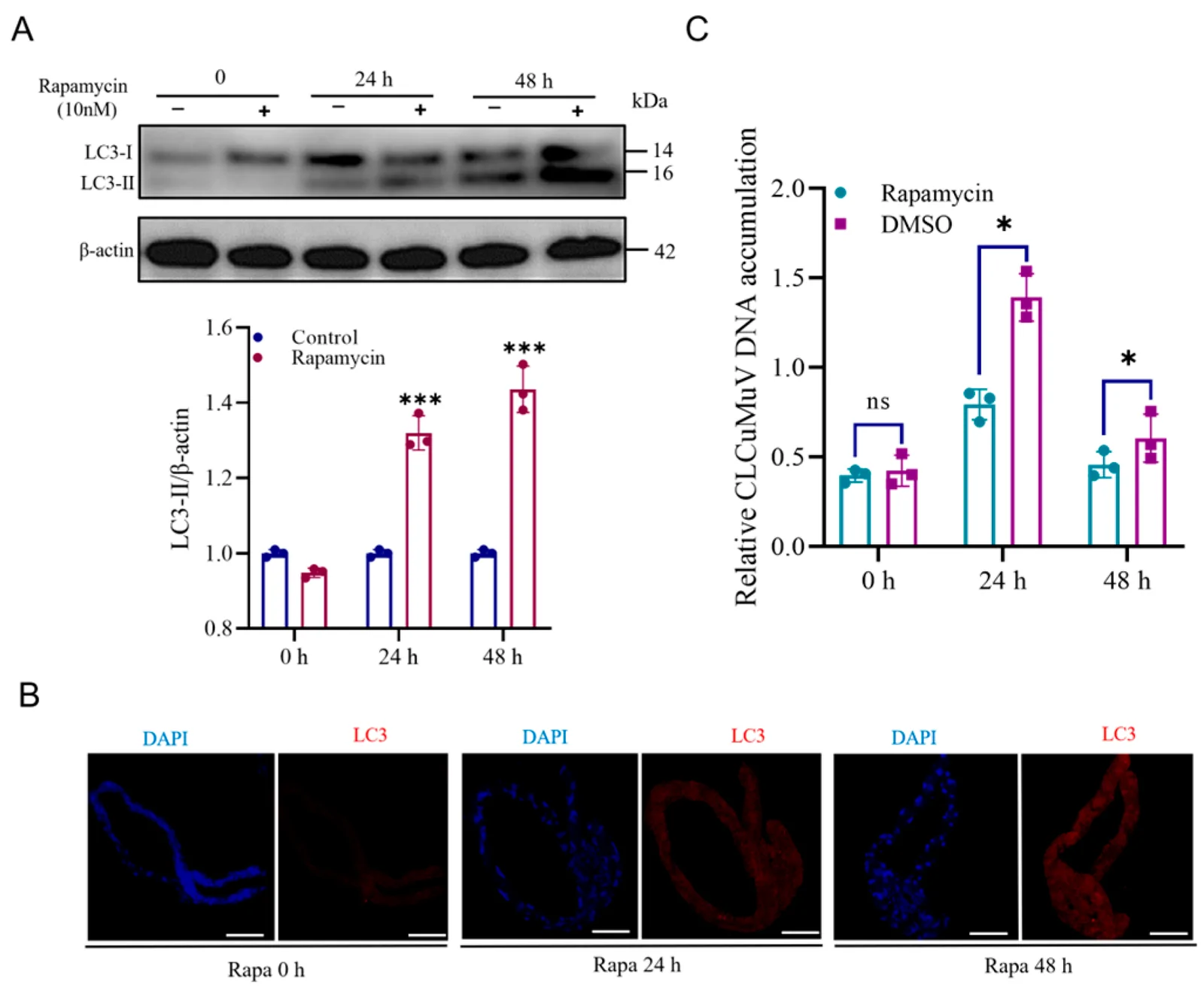
Rapamycin-induced activation of autophagy limits CLCuMuV DNA accumulation in *Bemisia tabaci*. (**A**) Effect of rapamycin treatment on the conversion of LC3-I to LC3-II in whiteflies. The bar graph below shows the quantitative ratio of LC3-II/β-actin based on band intensities. (**B**) Immunofluorescence microscopy showing the distribution and punctate accumulation of LC3 (red) in whiteflies after rapamycin treatment. Nuclei were stained with DAPI (blue). Scale bars are indicated in the panels. (**C**) Effect of rapamycin-induced autophagy on the relative accumulation of CLCuMuV DNA in whiteflies. Statistical significance was analyzed with Student’s *t*-test; “ns” indicates no significant difference, while asterisks indicate * *p* < 0.05, *** *p* < 0.001.

To further assess the role of autophagy as an antiviral defense in *B. tabaci*, we investigated whether artificially enhancing autophagy would conversely reduce viral accumulation. Whiteflies were treated with rapamycin (10 nM), a well-established specific inducer of autophagy. First, the activation of autophagy was verified at the protein level. Western blot analysis revealed that the conversion of LC3-I to LC3-II was significantly increased in the rapamycin-treated group at 24 h and 48 h post-virus acquisition compared to the control group, evidenced by the elevated LC3-II/β-actin ratio (Figure 3A). This biochemical activation was further confirmed by immunofluorescence microscopy. As shown in Figure 3B, while a diffuse LC3 signal was observed in untreated controls, abundant punctate LC3-positive aggregates were prominently formed in the cytoplasm at 24 and 48 h following rapamycin treatment, indicative of increased autophagosome formation.

Subsequently, we assessed the impact of induced autophagy on viral proliferation. Whiteflies were exposed to rapamycin for 24 h and then transferred to virus-infected cotton plants for 0, 24, or 48 h of virus acquisition. RT-qPCR analysis demonstrated that treatment with rapamycin decreased viral DNA accumulation by 46.4% and 33.3% relative to control levels at 24 and 48 h post-acquisition, respectively (Figure 3C). No significant difference in viral load was observed at the initial 0 h time point (Figure 3C). Together with the autophagy inhibition experiments, these results provided evidence that rapamycin treatment was associated with reduced CLCuMuV DNA accumulation, further supporting the conclusion that the CLCuMuV-induced autophagic response functions as a host antiviral defense mechanism in *B. tabaci*.

## 4. Discussion

Autophagy is a conserved degradation pathway that maintains cellular homeostasis and mediates antiviral defense in diverse insect–virus systems [18,30,31]. However, whether this pathway contributes to the immune response of the MED cryptic species of *B. tabaci* against CLCuMuV, a virus that persists in but is not transmitted by this vector, has remained unclear. In this study, we demonstrated that CLCuMuV acquisition activated autophagy in the MED *B. tabaci,* and that this response restricts viral accumulation, as evidenced by both loss- and gain-of-function assays.

The requirement of *Atg3* and *Atg9* for this antiviral effect is consistent with their conserved roles in autophagosome biogenesis. ATG3 acts as an E2-like enzyme mediating LC3 lipidation, while ATG9 is the only transmembrane ATG protein involved in membrane supply during autophagosome formation [32,33,34]. Silencing either gene significantly increased CLCuMuV DNA accumulation, suggesting that a functional autophagy pathway is necessary to constrain viral load. Similar observations have been reported in other insect–virus systems. In the MEAM1 cryptic species of *B. tabaci*, autophagy induced by TYLCV restricts viral accumulation through degradation of coat protein and genomic DNA, and silencing *Atg3* or *Atg9* similarly elevates viral load [12]. In the white-backed planthopper *Sogatella furcifera*, knockdown of SfATG3 or SfATG9 promotes propagation and transmission of Southern rice black-streaked dwarf virus [35]. These studies support the view that *Atg3*/*Atg9*-dependent autophagy represents a conserved antiviral mechanism in insect vectors. Pharmacological interventions reinforced this conclusion. Blocking autophagic flux with bafilomycin A1, which inhibits autophagosome–lysosome fusion [36], led to LC3-II accumulation and increased CLCuMuV DNA levels, confirming that complete autophagic degradation, rather than merely autophagosome formation, is required for viral restriction. Conversely, rapamycin-induced autophagy reduced viral accumulation. Together, these reciprocal results provide compelling evidence that the autophagy pathway negatively regulates CLCuMuV persistence in MED whiteflies. Nevertheless, since the present study focused solely on DNA-A accumulation without betasatellite quantification, whether betasatellites contribute to viral accumulation or vector–virus interactions in this system remains an open question for future studies.

A key finding of this study is that although autophagy modulates CLCuMuV accumulation, it does not enable MED whiteflies to transmit the virus. To directly test whether the increased viral load resulting from autophagy inhibition could overcome the transmission barrier, we performed inoculation assays using *Atg3*- and *Atg9*-silenced MED whiteflies on healthy cotton plants (*n* = 15 per treatment, three independent replicates). Despite significantly elevated viral DNA levels in these knockdown insects (Figure 2B), no whitefly-transmitted infection was detected in any of the recipient plants at 30 days post-inoculation (Table S1). Persistent-circulative transmission of begomoviruses requires a series of sequential steps: acquisition from phloem, midgut epithelial crossing, hemolymph circulation, salivary gland invasion, and release with saliva during feeding [37,38]. Autophagy may primarily influence viral retention or degradation in gut tissues, but successful transmission depends on whether virions can efficiently cross these tissue barriers and reach the salivary glands in a competent form [39]. In MED whiteflies, the major restriction likely operates at one or more of these barriers, and this barrier cannot be overcome simply by modulating autophagic activity or increasing total viral load.

This dissociation between viral accumulation and transmission distinguishes the MED–CLCuMuV system from the TYLCV–MEAM1 and CLCuMuV–AsiaII7 systems, where autophagy manipulation directly affects both viral accumulation and transmission efficiency [12,29]. In vectors, changes in viral load can translate into altered transmission efficiency; in non-vectors such as MED for CLCuMuV, viral accumulation can be modified without conferring transmissibility. Thus, the relationship between autophagy and transmission is contingent upon vector competence, and autophagy is not the sole or primary determinant of whether a whitefly cryptic species can transmit a given begomovirus. Future study should focus on pro-vector factors—receptors, trafficking components, or salivary proteins—that actively enable viral transmission.

The antiviral role of autophagy against CLCuMuV is not limited to insect vectors. In plants, autophagy has been shown to restrict geminivirus infection by targeting viral proteins for degradation [40,41,42]. For example, CLCuMuV βC1 bound to GAPCs and disrupted the interaction between GAPCs and autophagy-related protein 3, and is degraded through the autophagy pathway; disruption of this interaction results in increased viral DNA accumulation and more severe symptoms [43]. These observations, together with our findings, suggest that autophagy acts as a conserved defensive mechanism against CLCuMuV in both plant hosts and whiteflies. However, the viral substrates recognized by the autophagic machinery in insect vectors—whether coat protein, genomic DNA, intact virions, or virus-containing vesicles—remain to be identified. Future studies utilizing protein degradation assays and ultrastructural analyses will be necessary to elucidate the mechanistic of autophagy restricts CLCuMuV accumulation in AsiaII7 whiteflies.

## 5. Conclusions

In conclusion, our study shows that CLCuMuV acquisition activates autophagy in MED cryptic species of *B. tabaci* and that this response restricts viral accumulation within the insect body. Furthermore, CLCuMuV accumulation and transmission are governed by distinct mechanisms; autophagy is not the determinant of vector competence in this system.

## Figures and Tables

**Table 1:** Primers used in this study.

Primer Name	Primer Sequence (5′ → 3′)	Purpose
CLCuMuV CP	F: CAGGAAGCAGGAAAATACGAGA R: TGGCAGTCCAACACAAAATACG	qPCR
*Atg101*	F: CAGCGCAATTGGTCTTCAGC R: AGTGAGAAGGTCACCCACCT	
*Atg16-1*	F: CACATTCGGTAGAGTTAGTTTCGG R: CGGCATACACTTCTCCATCGT	
*Atg3*	F: CCAGATTGTCTCCAGCAGCA R: CGTTTAAGGGAACAGCACTTG	
*Atg5*	F: AGGTGAGAGCGAGTTCAAGC R: TGTCCGGGTAACTCAAGTGC	
*Atg6*	F: GTGCCCCCTGTGCTTTAGAT R: CGCAGCTCGTTGTTTGTTCA	
*Atg8*	F: GTCCTTGTGCTCCTCGTAGATG R: CTGACCGTCGGGCAGTTC	
*Atg9*	F: AGGGTTCCTGGTTCACGC R: TTGCCATCATTAACTTTCTGCT	
*Atg12*	F: TCAAAGCCACTGGAAACGC R: TCTGGTCTGGAGCAGGAGC	
*β-actin*	F: TCTTCCAGCCATCCTTCTTG R: CGGTGATTTCCTTCTGCATT	
*dsAtg3*	F: GCCAGTGGTGATGAATCAAG R: CCCTATCTTCAAGCCCTGAG	RNAi
*dsAtg9*	F: TTGGATTCGTTCTTCATTCG R: CCACACATTGGTCTGTTGGT	
*dsGfp*	F: AAGGGCGAGGAGCTGTTCACCG R: CAGCAGGACCATGTGATCGCGC	

## Data Availability

The data that support the findings of this study are available from the corresponding author upon reasonable request.

## Supplementary Materials

The following supporting information can be downloaded at: https://www.mdpi.com/article/10.3390/insects17080862/s1. Figure S1: PCR detection of the inoculated plants with MED whiteflies treated with *dsAtg3* and *dsAtg9* transmitting CLCuMuV; Table S1: Transmission efficiency of CLCuMuV to *Gossypium hirsutum* plants by MED whiteflies treated with *dsAtg3* and *dsAtg9*.
