# Supplementary material for: CLCuMuV-Induced Autophagy Restricts Viral Accumulation in the MED Cryptic Species of Bemisia tabaci
Source: Insects. 2026 Aug 19;17(8):862. doi: 10.3390/insects17080862 (PMC13513387; doi:10.3390/insects17080862)
Supplement: Supplementary file 1 [file insects-17-00862-s001.zip › insects-4496866-supplementary.pdf]

For the virus transmission experiment, adult whiteflies aged 7-8 days were fed diets supplemented with or without 0.25 μg/μL *dsAtgs* for a period of 24 hours. Approximately 200 adult whiteflies from each of the dsAtg-treated and each of control groups were then selected and permitted to feed on CLCuMuV-infected cotton plants. Following a 48-hour period, the viruliferous whiteflies were collected and placed into clip-cages on the third leaf from the bottom of uninfected cotton plants (Xinhai mian-21) at the 3-true leaf stage. After a 48-hour inoculation access period (IAP), the whiteflies were removed, and the plants were sprayed with imidacloprid at a concentration of 50 mg/L to eliminate any eggs. For each treatment group, 15 plants were used to assess virus transmission efficacy. Thirty days post-inoculation, leaves from each cotton plant were collected and analyzed via PCR [23] to determine the rate of virus infection. Additionally, the quantity of virus in each plant was quantified using qPCR. All the treatments were replicated 3 times.

Table S1. Transmission efficiency of CLCuMuV to *Gossypium hirsutum* plants by MED whiteflies treated with *dsAtg3* and *dsAtg9*

Treated	No. of tested plants	No. of diseased plants	Transmission efficiency (%)
dsGfp	15	0	0
dsAtg3	15	0	0
dsAtg9	15	0	0

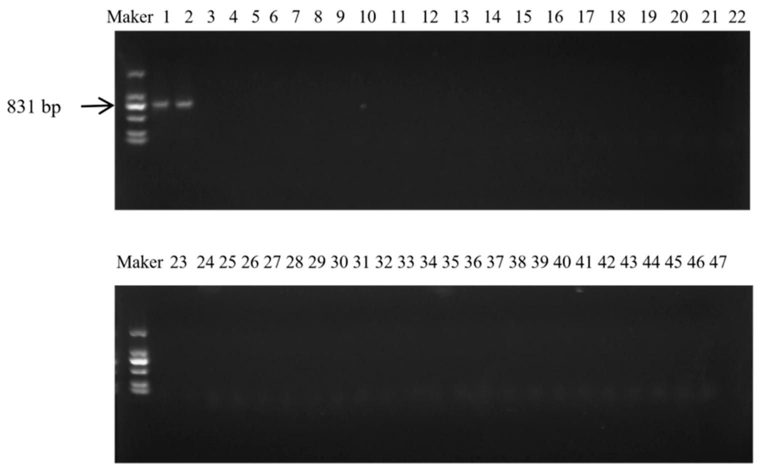

Figure S1. PCR detection of the inoculated plants with MED whiteflies treated with *dsAtg3 and dsAtg9* transmitting CLCuMuV

1-2: Diseased cotton plant infected by CLCuMuV. **3-17:** Plants inoculated with *dsGfp* treated whitefly transmitting CLCuMuV. **18-32:** Plants inoculated with *dsAtg3* treated whitefly transmitting CLCuMuV. **33-47:** Plants inoculated with *dsAtg9* treated whitefly transmitting CLCuMuV.
